# Autophagy characteristics of phytoestrogens in management and prevention of diseases: A narrative review of *in-vivo* and *in-vitro* studies

**DOI:** 10.5455/javar.2023.j683

**Published:** 2023-06-30

**Authors:** Safaa I. Khater, Maram Shalabi, Buthainah B. Alammash, Alaa I. Alrais, Doaa Al-ahmadi, Leena S. Alqahtani, Tarek Khamis, Sahar Abdelaziz, Khalifa Aldawy

**Affiliations:** 1 Department of Biochemistry, Faculty of Veterinary Medicine, Zagazig University, Zagazig, Egypt; 2 King Fahad Hospital, Ministry of Health, Medina, Saudi Arabia; 3 Maternity and Children Hospital (MCH), Ministry of Health, Medina, Saudi Arabia; 4 Department of Biochemistry, College of Science, University of Jeddah, Jeddah 23445, Saudi Arabia; 5 Department of Pharmacology, Laboratory of Biotechnology, Faculty of Veterinary Medicine, Zagazig University, Zagazig, Egypt; 6 Department of Pharmacognosy, Faculty of Pharmacy, Zagazig University, Zagazig, Egypt

**Keywords:** AMP-activated protein kinase (AMPK), autophagy, cancer, coumestans, isoflavones, lignans, phytoestrogens, stilbenes

## Abstract

Phytoestrogens are non-steroid polyphenolic materials present in 300 plants. Regarding their structural similarities to estradiol, phytoestrogens attach to estrogen receptors and display anti- or pro-estrogenic activities. This review explored phytoestrogens’ potential advantages and autophagy properties in light of their future application for disease management, highlighting how phytoestrogens could modulate autophagy. Research has examined the prospective benefits of phytoestrogens for the anticipation and management of various conditions, including signs of menopause, tumors, skin deterioration, osteoporosis, heart disease, neurodegenerative conditions, disorders of the immune system, and metabolic syndrome, owing to their therapeutic effects. As phytoestrogens can activate or inhibit autophagy, which has antioxidant, apoptotic, anti-mutagenic, anticancer, transcriptional, and genomic impacts on cancer and aging illnesses, phytoestrogens could influence diseases through the modulation of autophagy. The collaborative research on animal models, utilization of genetic techniques, and administration of pharmacologically active substances has indicated the possible therapeutic benefits of autophagy modulation in various illnesses. Further research is required to illustrate the pathways by which phytoestrogens modulate autophagy and the possible therapeutic effects on these diseases.

## Introduction

Phytoestrogens are natural herbal materials with structural similarities to 17-β-estradiol (E2), a mammalian estrogen [1]. These compounds are present in various plant-based nutrients, including fruits, vegetables, leguminous plants, and grains. Attributable to their structural similarities to estrogen, phytoestrogens combine with the estrogen receptors (ERs) in the human body, and elicit estrogen-like effects [2]. However, the attraction of phytoestrogens to ERs is generally inferior to that of endogenous estrogens [3,4]. Phytoestrogens have a range of potential health benefits. These compounds have been found to alleviate hot flashes in perimenopausal and menopausal women. Additionally, phytoestrogens may help prevent bone loss in aging women and alleviate menstrual issues [5]. There is some inconsistency in the impact of phytoestrogens on obesity induction and body composition, which suggests that the effect on adipose tissue distribution and metabolism is complicated and affected by additional variables [6].

Autophagy is a fundamental cellular mechanism contributing to the conservation of cellular homeostasis through the elimination of superfluous or defective intracellular constituents [7]. The process is crucial for the regular operation of cells and has been associated with various ailments, such as neurodegenerative conditions, malignancies, and cardiovascular disorders [8].

Recent literature has addressed the potential function of phytoestrogens in modulating autophagy and their potential therapeutic effects in various diseases. For example, studies have shown that phytoestrogens such as genistein protect cultured neuronal cells in a brain ischemia model through autophagy modulation [9]. Phytoestrogens have the potential to exert a dual effect on the regulation of autophagy. Specifically, they can act as contributing factors to autophagy initiation when autophagy serves as a mechanism for neuroprotection and promoting survival [10]. Conversely, phytoestrogens act as inhibiting agents of autophagy initiation when the autophagy process is a mechanism for promoting cell death. These conclusions propose that phytoestrogens have beneficial outcomes for brain ischemia based on their ability to modulate autophagy [11]. In addition to their potential neuroprotective properties, phytoestrogens also showed valuable effects in other diseases where autophagy plays a role [10]. For example, phytoestrogens have anti-cancer properties and can regulate vital biological processes in different types of cancer [12–14]. However, the mechanism by which phytoestrogens could modulate autophagy is still unknown, and the data included in the literature mostly address cancer studies with no previous reviews collecting the available data. Thus, phytoestrogens and their autophagy effects are debated regarding their latent use in managing conditions, emphasizing managing and protecting against cancer using the autophagy pathway and illustrating the probable modes of action of phytoestrogens on autophagy in the management of diseases.

## Phytoestrogens

### Sources and types of phytoestrogens

Red clover and other legumes, including soy, contain isoflavones. Soybean isoflavones, including biochanin A (BCA), genistein, formononetin, daidzein, and glycitein, are the most important sources of phytoestrogens [15]. Isoflavone phytoestrogens are the most thoroughly investigated. Isoflavones provide health benefits if consumed at 40–70 mg/day [16,17]. Grapes and nuts contain resveratrol, which is considered the most prevalent stilbene. Broccoli, nuts, spinach, and cabbage are all sources of estrogenic activity. Linseed, wheat, peanuts, berries, fruits, plants, coffee, and black tea all contain lignans. Enterolactone, an estrogenic and readily absorbed lignan, may be produced by intestinal microorganisms from matairesinol. Digestive bacteria in the intestinal tract metabolize phytoestrogens, which are digested and covalently linked by the liver. In addition, phytoestrogens are eliminated in the urine after circulating in the blood plasma. Structures comparable to endogenous estradiol allow phytoestrogens to attach to estrogen’s alpha and beta receptors [18].

Estrogen showed varied effects on each kind of receptor, Alpha and Beta. Alpha ERs facilitated cell growth, whereas cell death was reduced by beta ERs [19]. The receptor first attached to the ligand in the cytoplasm to regulate gene expression and then traveled to the cell nucleus, which influenced DNA transcription or small RNA. It is thus possible to modulate all progressions affected by estrogen, such as activation of sex hormones, binding to globulin, and inhibition of aromatase by phytoestrogens [20].

### Potential effects of phytoestrogens on diseases

Numerous studies have examined the properties of different forms of phytoestrogens on diseases, including osteoporosis, cancer, liver steatosis, heart conditions, coronary diseases, and obesity (Table 1).

#### High blood pressure

It is accompanied by high amounts of endogenous estrogen in women of reproductive age. To counteract the effects of estrogen, lignans will compete with natural estrogen for ERs, thus lowering high blood pressure [39]. Endogenous estrogen synthesis by the ovaries decreases throughout menopause. In times of low estrogen levels, lignans act as a weak form of estrogen [40].

#### Anticancer activity

Aromatase inhibitory effects are also provided by isoflavones, lignans, and proteins, which block the cytochrome P450 enzymes responsible for testosterone conversion to estrogen. Breast, adrenal, and prostate cancers are linked to higher cytochrome P450 enzyme levels [41]; thus, phytoestrogens showed anticancer effects.

#### Antiapoptotic activity

Other biological effects from phytoestrogens do not need ERs. Serotonin, insulin-like growth factor, displays intracellular regulation of apoptosis function and cell cycle through attachment to reactive oxygen species (ROS), methylation of DNA, the transcription of nuclear factor-kappa, histone alteration, and the expression of RNA. Phytoestrogens’ antioxidant, anti-proliferative, anti-mutagenic, and antiangiogenic properties result from this property, and these properties may help people live healthier and longer lives [42].

#### Adipose tissue and obesity

They have a role in adipose tissue progress and circulation during obesity. Phytoestrogens can activate or hinder Peroxisome proliferator-activated receptors (PPAR) signaling in adipocytes, determining whether they are beneficial or detrimental to weight loss. Genistein and daidzein have pro-adipogenic properties due to their ability to activate PPAR, while resveratrol is an antagonist of PPAR [43,44]. Phytoestrogens can also affect adipose tissue browning, as brown adipose tissue has a higher metabolic activity than white adipose tissue and can help burn calories. Resveratrol can activate sirtuins implicated in adipose tissue browning [45]. Phytoestrogens could affect adipose tissue metabolism. Estrogen acts as a regulator of both the volume and metabolism of adipose tissue; accordingly, a decrease in the levels of estrogen through menopause is related to a rise in obesity and a shift in adipose tissue distribution. Phytoestrogens can assist in managing body weight by influencing the distribution of adipose tissue [46–48].

**Table 1.** The different types of phytoestrogens, their chemical names and structures, and their potential uses in the treatment of various diseases.

Type	Chemical name	Chemical structure	Potential uses in treatment	References
Isoflavone	Genistein	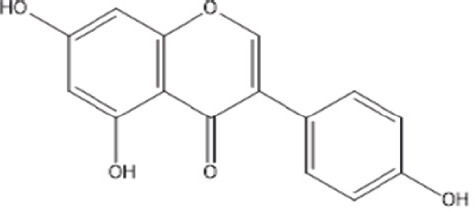	Induce apoptosis, inhibit cell proliferation, and differentiate cancer cells. The benefits of osteoporosis prevention include decreased likelihood of cancer progression, cardiovascular ailment, and relief from postmenopausal indicators	[21–24]
Isoflavone	Daidzein	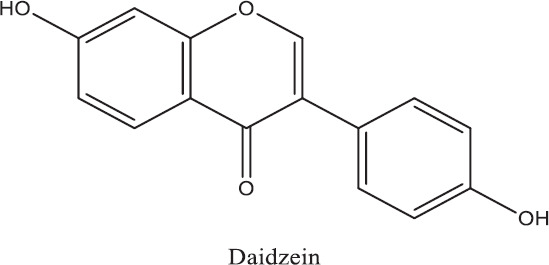	The inhibition and management of a diverse assortment of diseases, as coronary artery disease, diabetes, cancer, osteoporosis, skin conditions, and neurodegenerative disorders.	[25–27]
Coumestan	Coumestrol	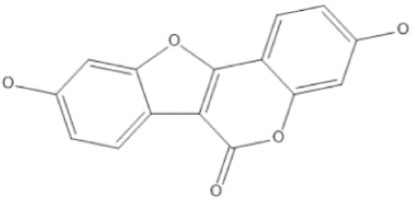	Anti-allergic, antioxidant, anti-adipogenic, anti-aging properties, anti-inflammatory, and anticancer effects.	[28–31]
Lignan	Enterolactone	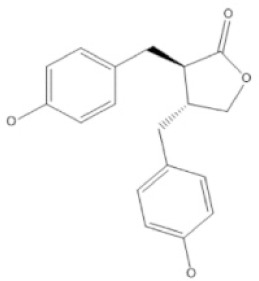	Mitigating the likelihood of specific cancer variants and enhancing cardiovascular well-being.	[32–38]
Lignan	Enterodiol	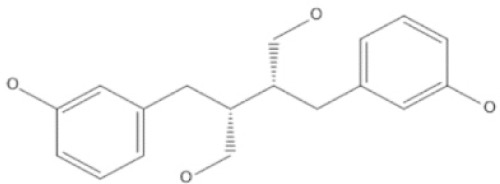		

#### Liver diseases

A study revealed that a diet rich in high-isoflavone soy protein isolate exhibited a protective effect against liver steatosis in obese Zucker rats. However, the administration of daidzein did not significantly advance liver steatosis levels, weight, calorie consumption, or serum adiponectin, and leptin levels in the rats [49]. The phosphorylation of AMPK in buffalo rat liver cells by genistein has demonstrated potential as a therapeutic intervention for fatty liver conditions [50].

#### Type II diabetes

Phytoestrogens can also affect blood sugar metabolism and type 2 diabetes. An improved lipid profile has been shown to result in an improvement in insulin tolerance and a decrease in plasma glucose and insulin scores in obese and lean rats [51,52].

#### Rheumatoid arthritis

A review published in 2021 examined the indications for the usage of phytoestrogens in the treatment of rheumatoid arthritis and found that phytoestrogens from diverse plant sources were capable of combating the signs of rheumatoid arthritis. Phytoestrogens reduce severe irritation and joint impairment by leveling several signaling and inflammatory compounds [53].

### Modes of phytoestrogen action

Phytoestrogens can act as agonists by activating the receptors and mimicking the effects of estrogen or other ligands. Phytoestrogens can also act as antagonists, which means they block the receptors and prevent the effects of estrogen or other ligands [54]. Phytoestrogens can also act as partial agonists, which means they have weaker effects than estrogen or other ligands. Phytoestrogens also function as selective estrogen receptor modulators because they have different properties in diverse cells or tissues [55].

The effects of phytoestrogens on these receptors can have various health implications, such as reproductive health, cardiovascular health, metabolic health, bone health, skin health, and immune system health. However, the exact mechanisms and outcomes of phytoestrogen action are still not fully understood and may depend on many factors, such as dose, duration, timing, source, individual variability, and interactions with other compounds [56].

#### Nuclear receptors modulations of transcriptional activity

ERs: phytoestrogens can induce or repress the transcription of ERs due to their parallel structure with E2. Also, the majority of phytoestrogens have low estrogenic activity when compared to E2; however, they have an advanced attaching property to ERβ more than ERα hence, elevated levels of phytoestrogens are needed to activate ERα in combination with ERβ [57] (Table 2).

PPARs: Phytoestrogens attach to PPARs, hinder downstream cellular processes, and affect their transcriptional activity, which is essential for the management of obesity and associated problems. PPAR activation by phytoestrogens could modulate adipogenesis and fat browning and enhance insulin sensitivity and blood glucose homeostasis, consequently being used to treat obesity among humans [58].

Aryl hydrocarbon receptor (AhR): AhR is triggered and regulates the immunological, reproductive, cardiovascular, and liver activities as they are capable of changing hormone pathways and connecting with steroid receptors [59].

Nuclear respiratory factors (Nrfs), also known as Nrfs, are transcription elements that play a vital function in controlling regulatory areas of target genes. Nrfs form dimers with the Musculoaponeurotic fibrosarcoma proteins (Maf) protein, resulting in a complex that attaches to the response element of oxidation (ARE). The engagement of Nrfs in the process activates antioxidative enzyme expression, which increases the defense of the cells against ROS toxicity [60].

#### Nuclear receptor-independent pathways

Phytoestrogens could bind to extranuclear ERs and the cell membrane, resulting in the following: Activation of protein kinases would phosphorylate some other transcription factors, promoting the transcriptional activity of phytoestrogens [61] and activation of G protein-coupled ERs [62]. Decreasing the ROS levels with stimulating antioxidants would stimulate AMPK [63,64]. Inhibition of protein tyrosine kinase inhibitors, thus regulating diverse signaling mechanisms in health and illness [65]. Inhibition of ribosomal S6 kinase and DNA topoisomerases (I, II) with activation of sirtuins, the nicotinamide adenine dinucleotide-dependent deacetylases, which control many genes expressions through alteration of transcription factors and histones [66]. Interfere with the function of estrogen-metabolizing enzymes, hence altering the biological effects of endogenous hormones [67]. Modify the epigenome: Epigenetic changes have been highlighted as effective mechanisms for controlling adipose tissue gene expression, including methylation of DNA and interference with microRNA [68]. Depending on the type and concentration of phytoestrogens, they can have different effects on these receptors.

**Table 2.** Mode of action of phytoestrogens through nuclear receptors modulations of transcriptional activity.

Receptor	Ligand	Effect
ERs	E2 or phytoestrogens	Activate or repress gene expression depending on the ligand and the target gene
PPARs	Phytoestrogens	Regulate adipogenesis and glucose homeostasis by modulating the countenance of gene’s protein sequence elaborate in lipid and carbohydrate digestion
AhR	steroid receptors	Affect immunological and reproductive systems by altering the translation of genes correlated with inflammation, cell cycle, and detoxification
Nrfs	Maf protein	Activate antioxidative enzyme expression by combining with the antioxidative response element (ARE) in the promoter part of the marked genes

*Estrogen receptors (ERs), 17-β-estradiol (E2), Antioxidative response element (ARE), Aryl hydrocarbon receptor (AhR), Nuclear respiratory factors (Nrfs), Musculoaponeurotic fibrosarcoma (Maf) proteins.

## Autophagy

**Figure 1.**
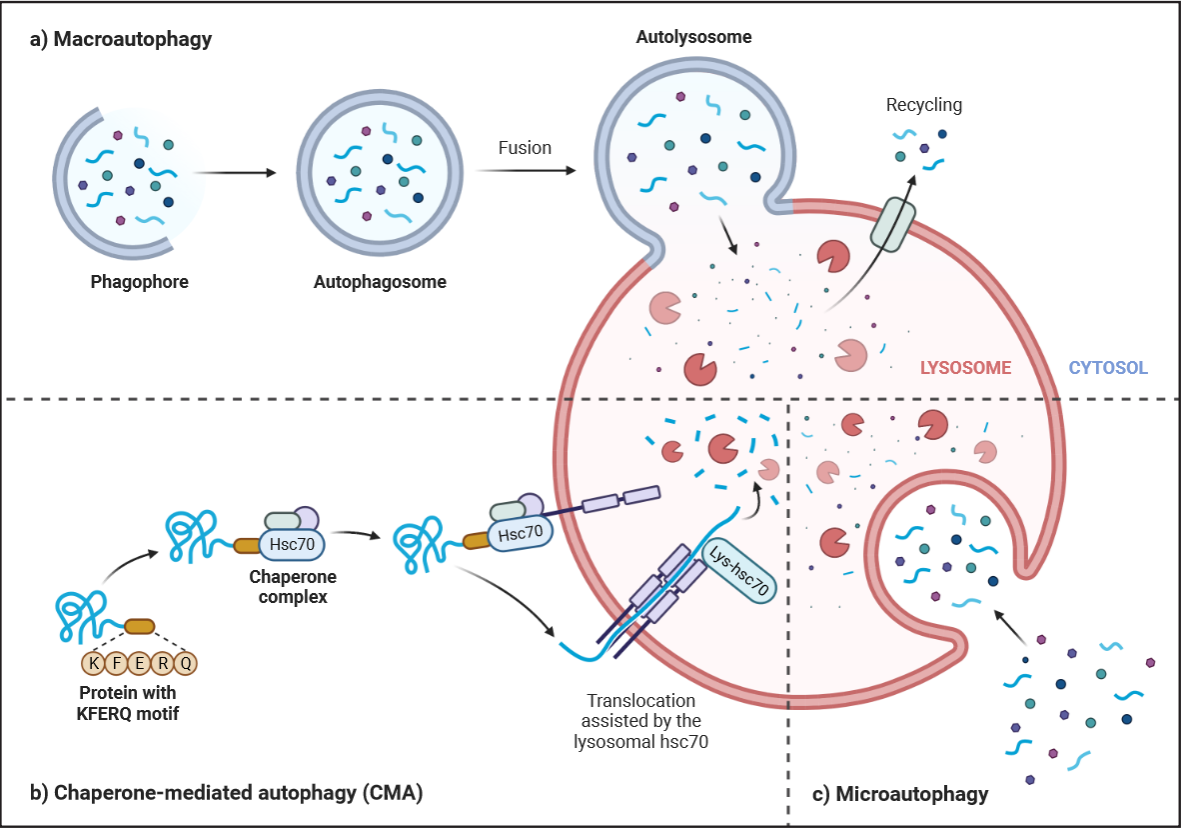
The kinds of autophagy among mammalian cells resulting in breakdown of cellular cargo and subsequent liberation of the resulting degraded materials into the cytosol, where they can be utilized by the cell. a) The process of macroautophagy involves the creation of new cytosolic vesicles with double membranes, known as autophagosomes, responsible for the sequestration and transportation of cellular materials to the lysosome. b) CMA facilitates the transportation of singular, unfolded proteins across the lysosomal membrane. c) Microautophagy entails the internalization of cellular compounds by the penetration of the lysosomal membrane. “Created with BioRender.com”

Autophagy is a catabolic procedure of lysosomes that has evolved. This process involves the degradation of damaged organelles and proteins that are subsequently utilized to synthesize ATP and new organelles [69]. There are three primary forms of autophagy: macroautophagy, microautophagy, and chaperone-mediated autophagy (CMA). Each type has its own unique characteristics, but all three result in the distribution of cellular material to the lysosome for degradation [70] (Fig. 1).

Microautophagy comprises the direct capture of cellular compounds by the lysosome through invaginations or protrusions of its membrane [71,72]. CMA, in contrast, uses chaperone proteins for identifying and transporting specific proteins transversely across the lysosomal membrane [73]. Macroautophagy encompasses the development of autophagosomes, double-membrane vesicles that impound cellular components and transfer them to the lysosome to be degraded [74,75].

### Autophagy in diseases

Autophagy is a fundamental cellular mechanism that facilitates the preservation of cellular homeostasis by eliminating superfluous or impaired cellular constituents [76]. Cellular homeostasis is a fundamental process that is indispensable for the proper functioning of cells and is involved in a multitude of physiological processes, such as progress, differentiation, and senescence [8,77]. Autophagy is a crucial pathway that safeguards the cells against diverse forms of stress, such as nutrient scarcity, hypoxia, and infection [8]. Autophagy aids in preserving the structural integrity of cellular organelles and mitigating the buildup of deleterious proteins and other hazardous substances by eliminating damaged or superfluous cellular components [78–80].

The primary cause of various human ailments, including cancer, neurodegenerative disorders, autoimmune, infectious ailments, circulatory diseases, obesity, type 2 diabetes, and deterioration, is aberrant alterations that either increase or reduce autophagy [81].

Studies have demonstrated that altered autophagy, whether increased or decreased, is present in obese individuals and experimental instances of obesity induced by diet or genetic susceptibility [82–84].

Autophagy is a highly effective mechanism for reducing inflammation, as it impedes the activation of inflammasomes and regulates the responses of type I interferons. Deretic et al. [85] state that it impacts the secretion of both inflammatory and antimicrobial mediators. Dysfunctional autophagy has been associated with the pathological buildup of cargo, which has been concerned with a diversity of human ailments, including neurodegenerative disorders, autoimmune and infectious conditions, and diverse types of cancer. Collective efforts in utilizing animal models, genetic tools, and pharmacologically active compounds have indicated the prospective therapeutic efficacy of autophagy modulation in various diseases [86].

Another study examined the effects of homocysteine on neural stem cell autophagy in animal and cell models of ischemic stroke. The study found that homocysteine reduced cell viability and enhanced nonapoptotic cell death in neural stem cells prone to oxygen-glucose deprivation or reoxygenation. The autophagy suppressor management partly inverted these effects, suggesting that homocysteine may cause excessive autophagy and facilitate its toxicity on neural stem cells in ischemic brains [87].

In addition to cancer and ischemic stroke, autophagy is involved in the development and progression of other diseases, as liver disease, myopathy, immune pathogen infection, and cardiovascular disease [88].

Furthermore, the multifaceted functions of autophagy in various domains, such as autoimmune diseases, infectious diseases caused by bacteria and viruses, neurodegeneration, tumor suppression and development, and brain growth, have been extensively discussed in the literature [89–91].

The autophagy pathway has been associated with various human diseases, thereby providing insights into novel therapeutic targets.

#### Phytoestrogens and Autophagy Effects

**Figure 2.**
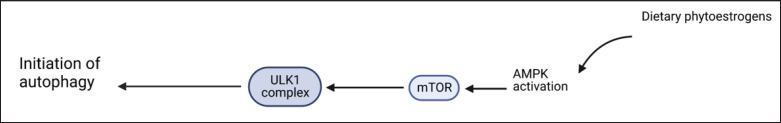
Dietary phytoestrogens activation of autophagy through phosphorylation of AMPK and mTOR pathway.

Recent studies have revealed a distinctive mechanism of phytoestrogen activity that entails the modulation of autophagy. The properties of phytoestrogens on autophagy initiation are contingent upon whether the activation of autophagy would result in cell death or survival, as they may either promote or impede the process in other diseases, not only cancer [92–95]. However, the majority of the studies in the literature entitled Outcomes of Phytoestrogens on Diseases were about the inhibition of cancer cells; the exact pathway by which these compounds can induce or inhibit autophagy remains a subject of debate. Some studies suggest that phytoestrogens can cause the phosphorylation of AMPK, leading to the activation of this enzyme and the inhibition of mTORC1, which in turn initiates autophagy. The previous pathway by which phytoestrogens initiate autophagy is illustrated in Figure 2.

Another way phytoestrogens can modulate autophagy is by activating transcription factor EB (TFEB). TFEB is a protein that adjusts the expression of genes concerned with autophagy and lysosomal function. When activated, TFEB can increase the expression of these genes, leading to an increase in autophagy. Table 3 provides a summary of some phytoestrogens’ effects on autophagy for the management of diseases.

### Effect of isoflavones on autophagy

Isoflavones are one class of phytoestrogens that have been shown to affect autophagy. BCA was investigated *in vivo* and *in vitro* to enhance the sensitivity of GBM cells to Temozolomide chemotherapy. The specific mechanism by which BCA enhances this sensitivity is through the inhibition of autophagy [93].

Sorafenib’s anti-proliferative and apoptotic actions can be enhanced by Bioincin-A, an isoflavone that inhibits the G0/G1 and pre-G cell cycle stages and reduces cyclin D1 protein levels in HCC cells [96].

The anticancer characteristics of genistein polyphenol could target diverse cancers. The stimulation of the oncogenic survival mechanism in NSCLC is dependent on the nuclear receptor co-repressor (N-CoR) misfolding that can be inhibited by genistein. According to Ali et al. [98], genistein was also linked to the heat shock cognate 70 kDa protein (HSC70), an autophagy molecular chaperone.

MCF-7 human breast cancer cells were used by Prietsch et al. [99] to observe the anti-proliferative properties of genistein. The research specified that the anticancer action of genistein was mediated via free radical production, the elevation of the BAX/Bcl-2 percentage, and the suppression of survivin, which finally led to the activation of autophagy and apoptosis. Although the research did not establish the precise function of autophagy, LC3-II immunostaining of breast cancer cells with genistein demonstrated autophagy progression.

Several studies have shown that genistein may augment the anticancer properties of chemotherapeutic drugs (such as 5-fluorouracil, gemcitabine, cisplatin, and oxaliplatin) by altering the autophagy-apoptosis pathway. The combination of genistein with 5-FU may promote autophagy in malignant cells by drastically affecting the expression of Beclin1 and Bcl-2, the two key molecules that control autophagy [100]. In addition, chloroquine, a recognized autophagy inhibitor, was used to validate the activation of autophagic cell death among the treated group using 5-FU and genistein [100].

Also, when genistein blocks mTOR, TFEB becomes less phosphorylated and moves into the nucleus. This is linked to a significant rise in lysosomal activity and content in both non-cancerous and cancerous cells that have been treated. Therefore, genistein seems to be a promising therapy for lysosomal storage disorders and malignancies [101]. TFEB is a principal controller of lysosomal biosynthesis and a primary amplifier of autophagy-associated protein production. Genistein increases the overexpression of TFEB, which is a surprising finding [102].

Soy isoflavones reduce oxidative stress in the substantia nigra caused by ATR Mitogen-Activated Protein Kinase Malondialdehyde MDA buildup and GSH reduction and inflammation damage (high IL-6 and Tumor necrosis factor). While ATR boosted Bax expression, decreased antiapoptotic expression of Bcl-2, and enhanced the proapoptotic factor Bax. SI therapy reversed all of these effects, indicating that SI may suppress the ATR-induced death of DAergic neurons. Furthermore, ATR suppressed autophagy of the significant nigra through the downregulation of LC3-II and Beclin-1 and the upregulation of p62, while SI pretreatment restored these effects, suggesting autophagy activation. In the substantia nigra, ATR raised the levels of mTOR and decreased Brain Expressed X-linked 2 (BEX2) and phosphorylated S6 (p-S6) expression. These data imply the ability of SI to inhibit ATR-induced neuronal degeneration by triggering autophagy through an mTOR-dependent signaling mechanism [104].

Consumption of genistein had varying effects on breast cancer cells with differing ER/ER ratios [105]. The combination of genistein and tamoxifen enhanced the rate of autophagic cellular death. On divergence, a high percentage has a detrimental impact on anticancer therapy owing to a reduction in ROS formation, which is a primary pathway of cisplatin- and tamoxifen-treated cells [105].

**Table 3.** Effects of phytoestrogens on autophagy.

Type of phytoestrogen	Type of model	Effect on autophagy	References
BCA	Glioblastoma (GBM) (*In-vitro* and *In-vivo*)	BCA cooperates with specific residues of AMPK and disrupts autophagy by modulating the AMPK/ULK1 mechanism.	[93]
Biochanin-A	Hepatocellular carcinoma cells (*In-vitro*)	boosted the anti-proliferative and apoptotic properties of SOR and induction of autophagy	[96]
Biochanin-A	Steatosis (HepG2 cells) (*In-vitro*)	Triggering SIRT3/AMPK/ULK-1-mediated autophagy	[97]
Genistein	Non-Small Cell Lung Cancer (NSCLC) Cells (*In-vitro*)	Initiation of autophagy by degradation of misfolded N-CoR protein	[98]
Genistein	human breast MCF-7 cells (*In-vitro*)	altering the expression of proapoptotic elements and enzymes involved in oxidative stress	[99]
Genistein	MIA PaCa-2 human pancreatic cancer cells (*In-vitro*)	Induction of autophagy through a rise in the progression of both Light chain (LC3-II) and beclin-1	[100]
Genistein	Niemann-Pick type C disease (*In-vitro*)	enhanced the expression of lysosomal proteins and promoted autophagic flux in NPC1 patient fibroblasts. This was evidenced with a decline in p62 and a rise in LC3-II levels in these cells.	[101]
Genistein	Human dermal fibroblasts (HDFa) (*In-vitro*)	Induction of autophagy, enhancement of degradation of glycosaminoglycans, and lysosomal degradation.	[102]
Puerarin	hepatocellular carcinoma (HCC) cells (*In-vitro*)	Initiation of apoptosis and autophagy by MAPK Signaling mechanisms	[103]
Soybean isoflavones	Rat model of ATR-induced DAergic toxicity (*In-vivo*)	SI has a neuroprotective effect by promoting autophagy via a signaling pathway that is dependent on mTOR, thereby preventing the degeneration of neurons mediated by ATR.	[104]
Genistein	Breast cancer cells (*In-vitro*)	Upregulation of antioxidant enzymes, cytotoxic agents, and reduction of ROS production, and induction of autophagy or apoptosis.	[105]
Genistein	Mammary Tumors to Tamoxifen in Rats (*In-vivo*)	downregulation of autophagy-related genes (IRE1α, GRP78, Beclin-1 and ATF4)	[106]
Genistein	Huntington’s Disease (HD) (*In-vitro*)	Treating HD cell model and increased viability of the cells through stimulating autophagy	[107]
Genistein	High glucose (HG)-treated podocyte model (*In-vitro*)	Genistein and Myd88 activate autophagy through inhibition of mTOR	[108]
Genistein	Hepatic stellate cell (HSC) (*In-vitro*)	Decreased LC3-II levels and boosted p62 expression, thus activating autophagy	[109]
Silibinin	DLBCL cell lines (*In-vitro* and *In-vivo*)	Initiated apoptosis and autophagy showing antitumor effects	[110]
Daidzein	Doxorubicin-induced cardiac injury (*In-vivo*)	Reduced autophagy and apoptosis by inhibiting the PI3K/Akt pathway	[111]
Vitexin 6	several tumor cell lines (*In-vitro*)	The activation of the Jun N-terminal kinase (JNK) pathway triggers autophagy and apoptosis	[112]
Arctigenin (ATG)	HEK293 cells (*In-vitro*) and Alzheimer’s disease model mice (*In-vivo*)	Autophagy is initiated by inhibiting the AKT/mTOR signaling mechanism and triggering the AMPK/Raptor procedure.	[113]
ATG	Human breast cell line (*In-vitro*)	Inhibits mTOR pathway and induction of autophagic cell death	[114]
ATG	HepG2 cells (*In-vitro*)	protects cells against ER stress by activating AMPK	[115]
ATG	HCC cells (*In-vitro*)	Induction of cell death via downregulation of PI3-K/Akt Signaling	[116]
ATG	naïve T cells (*In-vitro*) and DSS-induced mice (*In-vivo*)	Impeding the differentiation of Th1 and Th17 cells, suppressing the mTOR pathway, thus inducing autophagy	[117]
Combretum trachelogenin, fruticosum	HCT-116 human colon cancer (*In-vitro*)	Induced autophagic cell death by increasing LC3 stimulation and changing Beclin-1 expression	[118]

Research using a preclinical estrogen receptor-positive (ER+) breast cancer model revealed that lifetime genistein consumption decreases the likelihood of de novo, acquired tamoxifen resistance, and tumor relapse. The research also revealed that prepubescent and lifelong genistein users have enhanced sensitivity to tamoxifen [106]. The downregulation of PI3K-AKT signaling activation is connected with genistein’s oncosuppressive action [119].

Research studied the properties of genistein in a cellular model of HD comprising HEK-293 cells treated with a plasmid with a mutated HTT gene and found that the expression of mutated huntingtin and the quantity of aggregates were significantly reduced in the genistein-treated HD cell model. It resulted in increased viability of the cells through stimulating autophagy and removing the major pathogenic factor of HD [107].

Furthermore, a research paper investigated the effects of Genistein and Myd88 on autophagy in high glucose-induced renal podocytes. The study found that Genistein and Myd88 activate autophagy in these cells by inactivating mTOR signaling and stimulating LC3-II [108].

Genistein has been shown to inhibit HSC activation via PPAR-γ-regulated autophagy, which decreased the levels of LC3-II and boosted the levels of p62 in genistein-treated HSC-T6 cells; this implies that the autophagy pathway is being activated [109].

Silibinin, a natural estrogen receptor beta agonist, induced cell death and autophagy (*in vivo* and *in vitro*) and reduced tumor volume in vivo in Diffuse Large B-cell Lymphoma (DLBCL); thus, silibinin has the potential to be a therapeutic agent for the treatment of DLBCL [110].

Daidzein is a soy isoflavone that has shown protective effects against doxorubicin-induced cardiac injury in rats, as pretreatment with a low quantity significantly improved cardiac function and relieved histopathological deterioration of cardiomyocytes alleviated by doxorubicin. It decreased the protein expression of LC3 II, Bax, p-Akt, and cleaved caspase3, while enhancing cyclin D1 and Bcl-2, decreased apoptosis and autophagy by downregulating the PI3K/Akt pathway, thus defending the hearts from doxorubicin-induced cardiac damage [111].

Puerarin, another isoflavone studied by Zhang et al. [103], caused SMMC-7721 cells to undergo programmed cell death by causing depletion of MMP and the generation of ROS. Using a mitochondria-dependent mechanism, this isoflavone has anticancer properties.

### Lignans effects on autophagy

Lignans are another class of phytoestrogens that have demonstrated effects on autophagy. Lignans are plant-complex polyphenolic antioxidants. Many dietary lignans are transformed by intestinal bacteria into enterolignans, bioactive mammalian metabolites [120]. Lignans constitute an important group of polyphenolic compounds that can induce growth inhibition and apoptosis or autophagy-mediated cell death in cancer cells while exhibiting minimal cytotoxicity towards non-transformed cells [121]. Consuming lignan has been connected to the prevention of diverse forms of cancer via the modification of multiple signaling molecules and pathways. Anticancer lignans constitute a crucial category of polyphenolic compounds that induce growth inhibition and apoptosis, or autophagy-modulated cancer cell death, while exhibiting minimal cytotoxicity towards non-transformed cells. The aforementioned naturally occurring chemicals can inhibit the process of carcinogenesis, the growth of tumors, and the spread of cancer cells to other parts of the body. Research suggests that natural lignans, such as magnolol and honokiol, exhibit remarkable anticancer properties against several forms of tumors [122].

The compound Lignan Vitexin 6 (VB6) induced autophagy and programmed cell death in various types of cancer cells. The study discovered relationships between VB6-induced apoptosis, activation of Bax, cleavage of caspase-3, and decreased expression of Bcl-2 that depended on both time and concentration. Also, LC3-II and Beclin-1 levels (markers for autophagy) exhibited a gradual increase subsequent to VB6 treatment. Zhou et al. [112] reported a rise in JNK phosphorylation, P-C-Jun, and P-Bcl-2 expression subsequent to VB6 therapy.

The mTOR pathway, which is an integral part of cellular development and proliferation, can be inhibited by lignans, a form of phytoestrogen that has been discovered to have this effect. ATG lignans have anti-colitis actions in immune cells that produce endoplasmic reticulum stress and the death of liver cancer cells, and they accelerate the clearance of beta-amyloid in Alzheimer’s disease [113]. According to Maxwell et al. [114], lignans were able to block mTOR’s pathway effector molecules in human ER-positive MCF-7 breast cancer cells, which led to autophagy-mediated cellular death and downregulation of ER.

Also, ATG is a natural component that has protective properties against ER stress in vitro. According to a scholarly article, ATG demonstrated concentration-dependent inhibition of apoptosis and unfolded protein response (UPR) in cells exposed to brefeldin A, an endoplasmic reticulum (ER) stress inducer. The study found that ATG had a notable effect on reducing cellular protein synthesis by inducing mTOR signaling and eEF2 activity. This effect was observed to be partially reversible by the use of RNA interference to silence AMPKα1. The reduction of intracellular ATP levels and activation of AMPK were observed upon ATG administration, which was attributed to the inhibition of complex I-mediated respiration. The inhibitory properties of ATG on ER stress were restored by pretreating cells via the AMPK inhibitor compound C [115].

ATG showed alleviating properties on HCC through downregulation of PI3-K/Akt signaling [116] and anti-colitis effectiveness on the differentiation of Th1 and Th17 cells through an mTORC1-dependent process [117]. Trachelogenin lignans constantly enhance autophagic cell destruction, intracellular vacuolization, and the generation of autophagosomes [118]. This was accomplished by enhancing LC3 stimulation and changing Beclin-1 expression.

According to Jung et al. [123], inhibiting the mTOR pathway may lead to an increase in autophagy, a reduction in protein translation, cellular proliferation, and progress, which have the potential to have an effect on cancer. Also, it was discovered by Wu et al. [124] that lignans inhibit the activation of mTORC1 in Th17 cells via adhering to ER in these cells. Polyphenols found in high quantities in plant-based diets and beverages have been demonstrated in a variety of studies to successfully inhibit autophagy in diverse forms of cancer [125].

### Stilbenes effect on autophagy

Stilbenes, a class of phenolic compounds, have been demonstrated to influence autophagy in different types of diseases. Research has indicated that stilbenes can induce endoplasmic reticulum stress, block Wnt processing, and stimulate autophagy in acute lymphoblastic leukemia cells expressing Wnt16 [126]. Additionally, plant phenols, including stilbenes, have been shown to exhibit antioxidant properties and protect cells from oxidative stress [127]. These compounds may also exert a protective effect through the stimulation of the Keap1/Nrf2/ARE redox-sensitive signaling pathway and by modifying autophagy [128].

## Conclusion

Phytoestrogens are naturally occurring herbal composites with various health benefits. Recent research has focused on their potential role in modulating autophagy and their potential therapeutic effects in various diseases where autophagy plays a role. The findings revealed that the protective properties of phytoestrogens necessitate autophagy induction while they exhibit AMPK activation, which modulates the mTOR pathway of autophagy. However, all the possible mechanisms by which phytoestrogens modulate autophagy are still unknown, but the above-mentioned studies have confirmed the implication of phytoestrogens in the regulation of autophagy, which is important for the management of various diseases. The findings’ summary suggests the following:

The previous research has not addressed the major pathways of phytoestrogens modulating autophagy.

The antioxidant, apoptotic, anti-inflammatory pathways and the ability of phytoestrogens to modify transcriptional factors and micro-RNA were weakly illustrated in the previous studies to alter autophagy.

This review has collected all the published possible pathways by which phytoestrogens can suppress or induce autophagy either by the phosphorylation of AMPK, inhibition of mTORC1 or activation of TFEB.

Further research is required to fully comprehend how phytoestrogens modulate autophagy and their potential therapeutic effects in various diseases.

Phytoestrogens can be used after extensive future studies as promising agents in the management and prevention of many diseases through the modulation of autophagy.

## Limitations

The present review has some limitations, including limited data on human studies; thus, it was excluded from the study. The limitation of data from the literature regarding the effect of phytoestrogens on autophagy to modulate other diseases than cancer and the improper illustration of their pathways.

## List of abbreviations

AhR, Aryl hydrocarbon receptor; AMPK, AMP-activated protein kinase; ARE, Antioxidative response element; E2, 17-β-estradiol;ERs, Estrogen receptors; HCC, Hepatocellular carcinoma; HDFa, Human dermal fibroblasts; IL-6, Interleukin-6; JNK, Jun N-terminal kinase;; LC3, Light chain 3; Maf, Musculoaponeurotic fibrosarcoma proteins; MAPK, Mitogen-Activated Protein Kinase; MDA, Malondialdehyde; mTOR, Mammalian target of rapamycin;; N-CoR, Nuclear receptor co-repressoR;; Nrfs, Nuclear respiratory factors NSCLC, Non-Small Cell Lung Cancer; P62, Ubiquitin-binding protein; PI3K, Phosphoinositide 3-kinase; PPARs, Peroxisome proliferator-activated receptors; P-S6, Phosphorylated S6 protein; ROS, Reactive oxygen species; TEEB, Transcription factor EB, Tumor necrosis factor;.
